# Inappropriate sinus tachycardia-induced cardiomyopathy with severe functional mitral regurgitation and successful treatment with ivabradine

**DOI:** 10.1016/j.jccase.2021.05.010

**Published:** 2021-06-20

**Authors:** Taiji Inamori, Kazuhisa Kodama, Yudai Tamura, Hideharu Okamatsu, Yukiko Sashida, Yoko Horibata, Eiji Taguchi, Koichi Nakao, Tomohiro Sakamoto

**Affiliations:** Division of Cardiology, Saiseikai Kumamoto Hospital Cardiovascular Center, Kumamoto-city, Japan

**Keywords:** Ivabradine, Inappropriate sinus tachycardia, Tachycardia-induced cardiomyopathy, Decompensated heart failure, Functional mitral regurgitation

## Abstract

Ivabradine increases stroke volume, but does not have a negative impact on blood pressure (BP). Thus, a patient with low BP can benefit from treatment with ivabradine. A 72-year-old Japanese woman with asthma and chronic bronchitis presented with dyspnea. Her heart rate (HR) was 126 beats per minute and an electrocardiogram showed sinus tachycardia. The chest X-ray showed cardiomegaly and pulmonary congestion. A transthoracic echocardiogram (TTE) showed reduced left ventricular ejection function (LVEF) and severe functional mitral regurgitation (MR). We diagnosed her with inappropriate sinus tachycardia (IST) and heart failure (HF) due to tachycardia-induced cardiomyopathy. After resolving the pulmonary congestion with diuretics, we administered a minimum dose of bisoprolol, which resulted in re-exacerbation of the HF. Because IST was persistent, we initiated treatment with ivabradine. As soon as ivabradine was started, the HR decreased, the BP gradually increased, and HF compensation was achieved. Bisoprolol was continued and losartan was started. In summary, we used ivabradine for a patient with tachycardia, low BP, a low LVEF, and severe MR. By optimizing the medical therapy, exercise tolerance improved and she was discharged. The serum brain natriuretic peptide was significantly reduced and TTE showed an improved LVEF and reduced MR.

<**Learning objective:** We managed a patient who had low blood pressure (BP) due to tachycardia, reduced left ventricular ejection function (LVEF), and severe mitral regulation (MR). In this case, ivabradine had a novel effect; specifically, heart rate was reduced and BP increased. As a result of the drug effects, we could prescribe a renin-angiotensin-system inhibitor. With optimal medical therapy, LVEF was restored and functional MR was reduced. In similar cases, ivabradine can be a key drug for medical therapy of heart failure.>

## Introduction

Recently, we have begun to use ivabradine in Japan. Ivabradine prolongs the diastolic period more than β-blockers [1,2] and does not have the negative lusitropic and α-adrenergic coronary vasoconstriction effect of β-blockers [3]. In addition, ivabradine reduces afterload with an improvement in arterial compliance [4]. All of the mechanisms increase stroke volume. We used ivabradine for a patient with tachycardia, low blood pressure (BP), reduced left ventricular ejection function (LVEF), and severe mitral regulation (MR). LVEF and MR were significantly improved by optimizing the medication.

## Case report

**Fig. 1:**
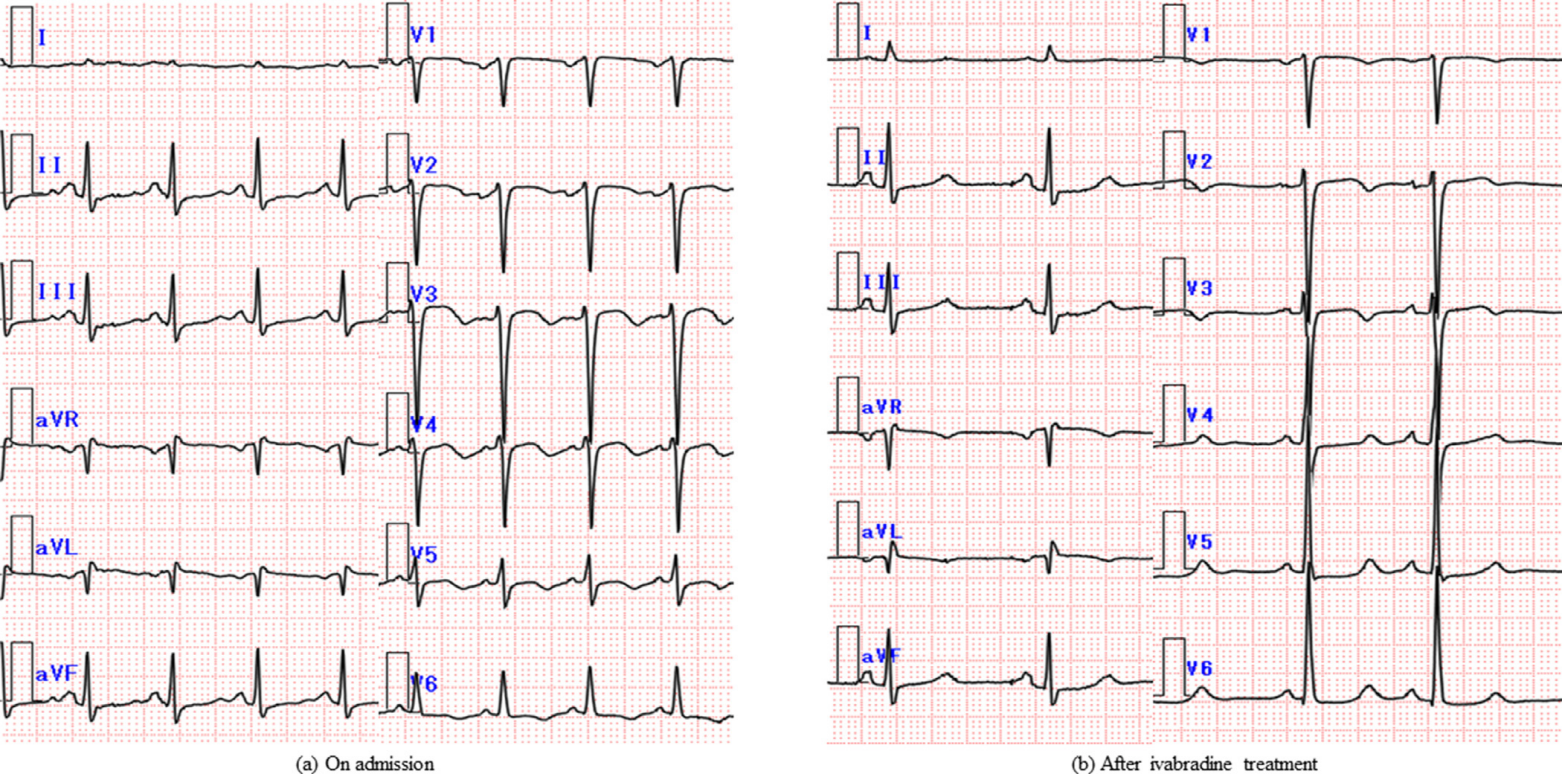
Electrocardiogram (ECG) changes. The ECG obtained at the time of admission showed that the patient had sinus tachycardia with a heart rate of 126 bpm (a). After ivabradine treatment, the ECG showed sinus rhythm with the heart rate reduced to 65 bpm (b).

A 72-year-old Japanese woman was admitted to our institute with complaints of dyspnea, shortness of breath, and palpitations which progressed over 6 hours. She had asthma and chronic bronchitis, but no history of cardiac disease. Her regular medications included salmeterol xinafoate / fluticasone propionate, montelukast, and erythromycin. Three years previously, she had a stroke and was started on cilostazol (100 mg twice a day) for secondary prevention. Her resting heart rate (HR) was approximately 70 beats per minute (bpm) before the cilostazol was started; however, an electrocardiogram (ECG) showed a sinus tachycardia with a HR of 120 bpm on admission. Initially, she was diagnosed with asthma and received intravenous methylprednisolone. Then, because there was little improvement, a cardiology consultation was requested. The ECG showed sinus tachycardia (Fig. 1a), and a chest X-ray (CXR) showed an enlarged cardiothoracic ratio (CTR; 56.7 %), and pulmonary congestion. On transthoracic echocardiogram (TTE), left ventricular end-diastolic diameter was 53 mm, LVEF was reduced to 35%, and severe functional MR was present. Six months before, she underwent a routine TTE, which was essentially normal beside the mild MR due to posterior commissure prolapse. Physical examination and blood testing showed no severe anemia, thyroid function abnormalities, or infection. Serum brain natriuretic peptide (BNP) was 1139.8 pg/ml. We therefore performed a coronary computed tomography to evaluate the cause of the cardiac dysfunction, but no coronary artery abnormalities were identified. Contrast-enhanced cardiac magnetic resonance imaging showed no evidence of wall thickening or late gadolinium enhancement. We suspected tachycardia-induced cardiomyopathy (TIC) as the cause of the cardiac malfunction. Based on her history, we considered cilostazol as a cause of tachycardia and changed cilostazol to clopidogrel. To improve congestion, we began diuretic therapy.

**Fig. 2:**
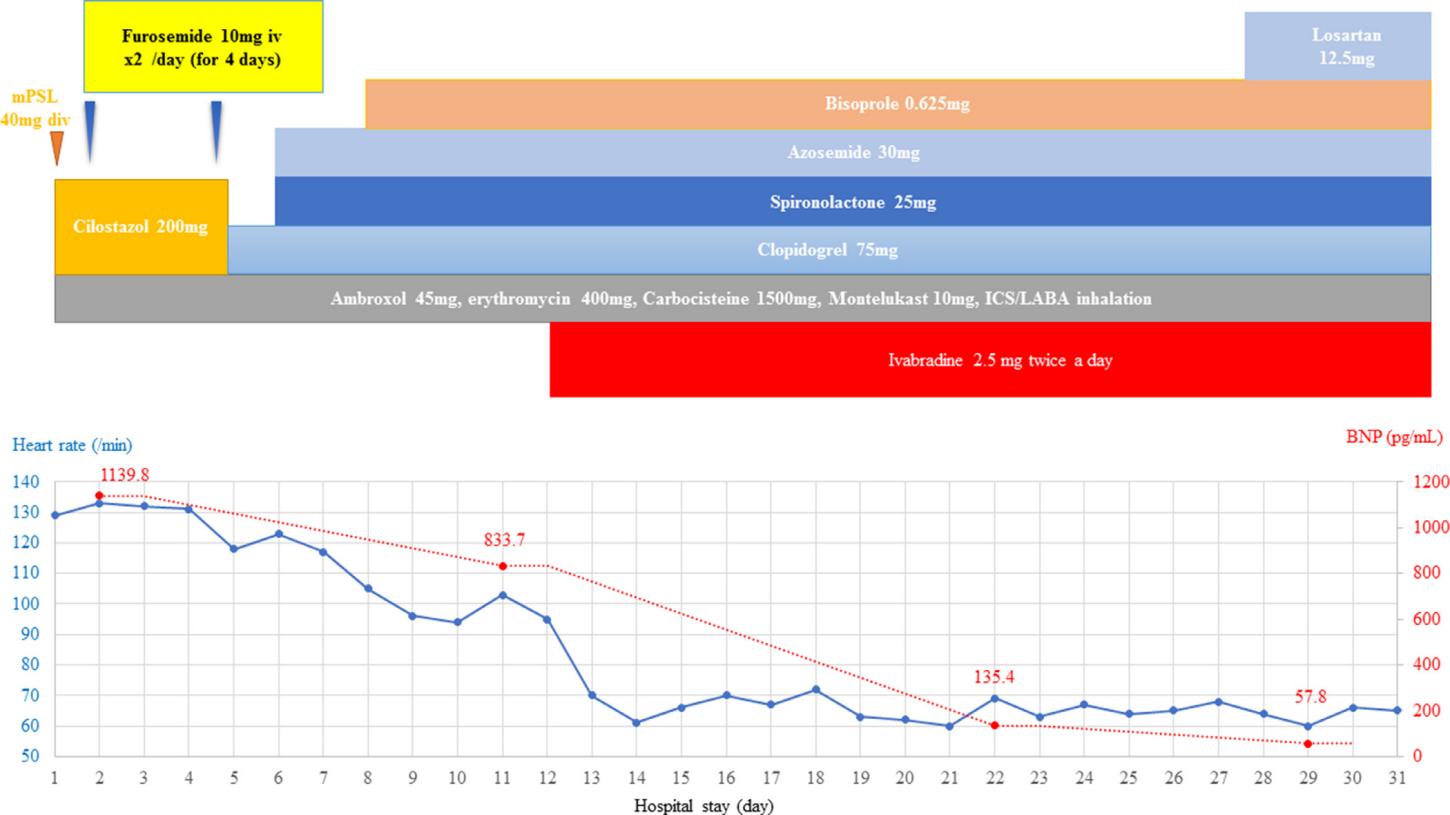
Hospital course.BNP, B-type natriuretic peptide; ICS, inhaled corticosteroid; LABA, long-acting beta 2 agonist; mPSL, methyl prednisolone.

Seven days after discontinuing cilostazol, the HR was approximately 110 bpm. The pulmonary congestion improved and CTR decreased to 50% with diuretics, so a low-dose β-blocker was added [bisoprolol (0.625 mg once a day)] for the purpose of controlling HR. The resting HR decreased to approximately 100 bpm, but there was a re-exacerbation of the pulmonary congestion and CTR increased to 53.5% on CXR. Thus, she did not tolerate β-blockers and a further increase in dose would be difficult. Therefore, we started ivabradine (2.5 mg twice a day) on the 12th hospital day to decrease the HR. Subsequently, the resting HR decreased from 100 bpm to 60 bpm, and the systolic BP increased from <80 mmHg to approximately 100 mmHg. The symptoms improved gradually and the urine output increased. The BNP improved from 1139.8 pg/ml to 135.4 pg/ml on the 23rd hospital day and the New York Heart Association functional classification improved to Class I. On the 25th hospital day, we added losartan, a renin-angiotensin-system inhibitor (RAS-I; 12.5 mg once a day). She was discharged on the 29th hospital day with a serum BNP of 57.8 pg/ml. The symptoms had completely resolved; LVEF also improved from 29% to 39%, and the MR was reduced. The clinical course is summarized in Fig. 2.

## Discussion

Fig. 2 In this case, the heart failure was decompensated due to TIC and functional MR. We initially thought the patient had drug-induced sinus tachycardia, which was caused by cilostazol. The duration of pharmacological effect of cilostazol is said to be up to 48 hours, but the tachycardia persisted even after cilostazol withdrawal. Although β-blockers are a favorable treatment for patients with inappropriate sinus tachycardia (IST), we could not achieve good control of IST with them. Another therapeutic option was ivabradine, calcium channel blockers, antiarrhythmic drugs, and radiofrequency ablation. Ivabradine prolongs the diastolic period more than β-blockers [1,2]. Ivabradine does not have the negative lusitropic and α-adrenergic coronary vasoconstriction effect of β-blockers [3]. In addition, ivabradine reduces afterload by improving arterial compliance [4]. All of the mechanisms increase stroke volume, and ivabradine does not have a negative impact on BP. Thus, we initiated ivabradine treatment to reduce HR. It is sometimes difficult to assume whether sinus tachycardia is necessary for patients with acute heart failure. However, we can calculate the optimal heart rate by using the deceleration time (DcT) of E-wave which is measured by the pulse Doppler echocardiography at the transmitral flow. The formula is that: (Ideal heart rate) = 93 – 0.13 * (DcT) [5]. In this case, we could estimate the ideal heart rate as 79 bpm on day 11. We started ivabradine on day 12 and heart rate decreased to around 70 bpm. The stroke volume estimated by Doppler was increased from 30 to 41 mL, and LVEF and MR improved. The BP gradually increased, then we started RAS-I, which further improved the heart failure.

**Fig. 3:**
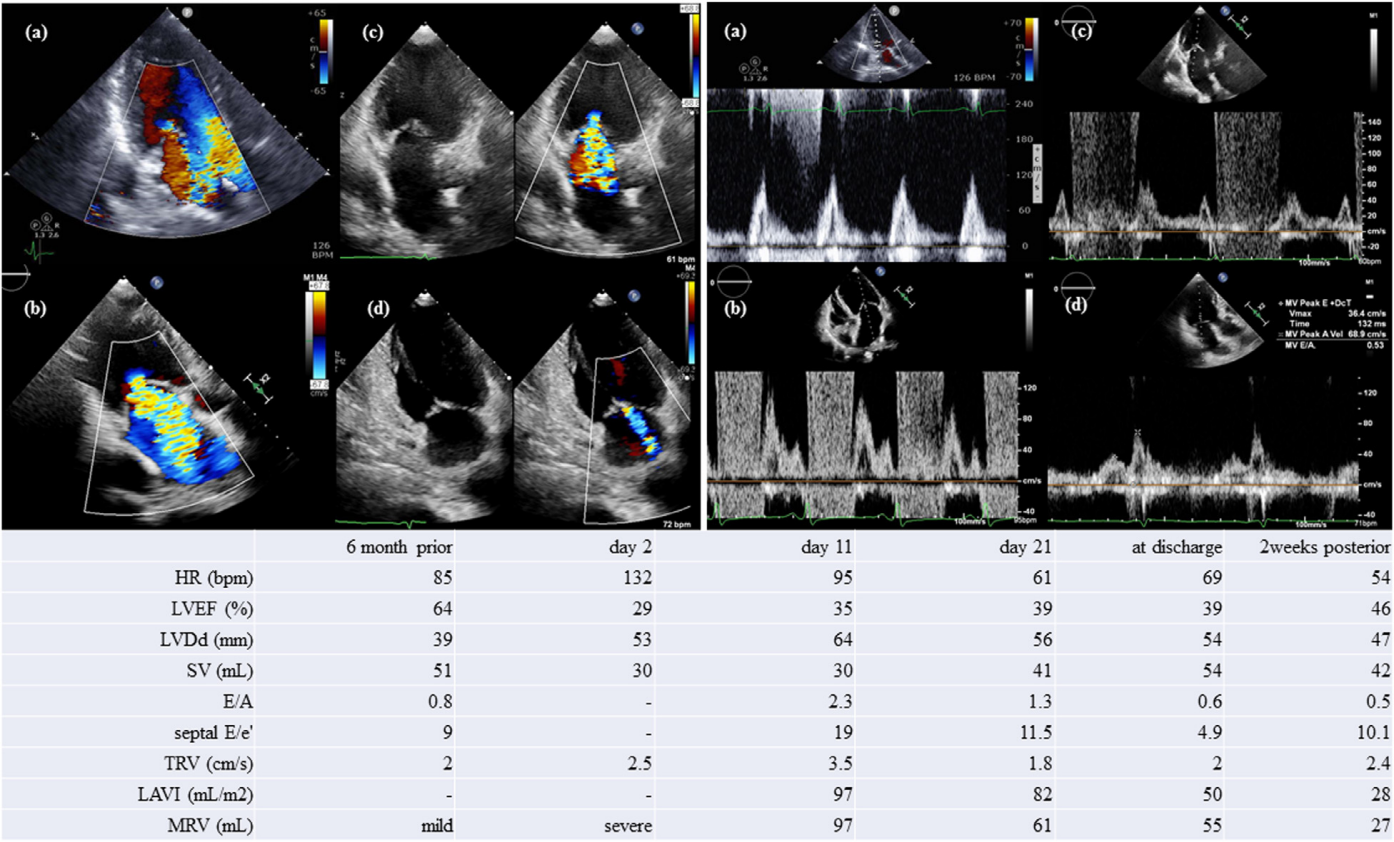
Transthoracic echocardiogram (TTE) changes. The chart shows the parameter of the TTE and her heart rate (HR) when she had had a TTE. The parameters include left ventricular ejection function (LVEF), left ventricular end-diastolic diameter (LVDd), stroke volume (SV) obtained by Doppler, early to late diastolic transmitral flow velocity (E/A), E to early diastolic mitral annular tissue velocity (E/e'), tricuspid regurgitant velocity (TRV), left atrial volume indexes (LAVI), and mitral regurgitation obtained by volumetric method (MRV). On day 2, mitral E and A waves fused due to tachycardia. The left panel shows color Doppler of mitral regurgitation. The right panel shows mitral inflow and mitral annular motion velocities. In both panels: (a) On day 2; (b) On day 11; (c) On day 21; (d) 2 weeks posterior to discharge.

For 8 weeks, 3 weeks after discharge, the LVEF improved from 29% to 46% and the MR improved from severe to mild; specifically, the MR volume decreased from 97 mL to 27 mL. In the case of tachycardiac heart failure with a reduced ejection fraction (HFrEF) and functional MR, ivabradine might play an important role. All of the TTE parameters thought necessary for evaluating hemodynamic status are shown in Fig. 3.

In this case, controlling HR and improving LVEF and MR contributed to the increase in BP, after which RAS-I treatment was begun. HFrEF patients usually have a low BP. Nevertheless, cardioprotective drugs, such as a β-blocker and RAS-I, are key drugs for HFrEF, but the lusitropic or vasodilation effect decreases the BP, thus we often have trouble using them. In contrast, ivabradine does not have a negative impact on BP, thus ivabradine can be started without concern. This case also showed that ivabradine might increase the BP, enabling an increase in cardioprotective drugs. We have shown that ivabradine improved the tachycardiac HFrEF and MR in a patient with a low BP. Further studies are warranted to evaluate the use of ivabradine treatment for such patients.

## Declaration of Competing Interest

None.
